# How to distinguish promotion, prevention, and treatment trials in public mental health: development and validation of the VErona-LUgano Tool (VELUT)

**DOI:** 10.1017/S2045796025100280

**Published:** 2025-11-12

**Authors:** Marianna Purgato, Emiliano Albanese, Alden L. Gross, Anna Maria Annoni, Ceren Acarturk, Camilla Cadorin, Mark J. D. Jordans, Crick Lund, Davide Papola, Eleonora Prina, Marit Sijbrandij, Manuela Silva, Federico Tedeschi, Wietse A. Tol, Corrado Barbui

**Affiliations:** 1 WHO Collaborating Centre for Research and Training in Mental Health and Service Evaluation, Department of Neurosciences, Biomedicine and Movement Sciences, Section of Psychiatry, University of Verona, Verona, Italy; 2 Cochrane Global Mental Health, University of Verona, Verona, Italy; 3 Institute of Public Health, Faculty of Biomedical Sciences, Università della Svizzera Italiana, Lugano, Switzerland; 4 Department of Psychiatry, University of Geneva, Geneva, Switzerland; 5 Department of Epidemiology, Johns Hopkins Bloomberg School of Public Health, Johns Hopkins University Center on Aging and Health, Baltimore, MD, USA; 6 Department of Psychology, Koc University, Istanbul, Türkiye; 7 Research and Development Department, War Child, Amsterdam, The Netherlands; 8 Amsterdam Institute for Social Science Research, University of Amsterdam, Amsterdam, The Netherlands; 9 Centre for Global Mental Health, Health Service and Population Research Department, Institute of Psychiatry, Psychology and Neuroscience, King’s College, London, UK; 10 Alan J Flisher Centre for Public Mental Health, Department of Psychiatry and Mental Health, University of Cape Town, Cape Town, South Africa; 11 Department of Clinical, Neuro- and Developmental Psychology, WHO Collaborating Center for Research and Dissemination of Psychological Interventions, Amsterdam Public Health Institute, Vrije Universiteit Amsterdam, Amsterdam, The Netherlands; 12 Lisbon Institute of Global Mental Health, Comprehensive Health Research Center, NOVA University of Lisbon, Lisbon, Portugal; 13 Section for Global Health, University of Copenhagen, Copenhagen, Denmark; 14 Athena Institute, Vrije Universiteit Amsterdam, Amsterdam, The Netherlands; 15 Arq National Psychotrauma Centre, Diemen, The Netherlands

**Keywords:** evidence synthesis, item response theory, mental health, prevention, promotion

## Abstract

**Background:**

Promoting mental health, preventing mental disorders and providing effective treatments are public health priorities. Randomized controlled trials (RCTs) frequently evaluate mental health and psychosocial support interventions to achieve one or more of these objectives. Distinguishing between RCTs focused on mental health promotion, prevention or treatment remains conceptually and methodologically challenging. No standardized tool exists to position RCTs along a promotion-to-treatment continuum in mental health. We aimed to develop and validate the VErona-LUgano Tool (VELUT) for distinguishing RCTs along the promotion-to-treatment continuum.

**Methods:**

An interdisciplinary tool development group (TDG) was established. The Population, Intervention, Comparison and Outcome framework was used to define key constructs. Items in the tool were devised, categorized and reduced through qualitative and quantitative methods. Finally, we performed a preliminary validation of the VELUT applying item response theory (IRT) using data from 180 RCTs.

**Results:**

The TDG generated 33 items for the initial version of the VELUT, reduced to 16 through review, cognitive interviews and psychometric analysis. Analyses of 180 RCTs using the 16-item tool showed high internal consistency (α = 0.94) and unidimensionality. Following item reduction and IRT, a final 8-item version was retained, and IRT models confirmed strong item discrimination for the 8 items and high scale reliability (marginal reliability >0.90 across most of the range of the scale), good response distribution, item performance and alignment with the Institute of Medicine (IOM) promotion-to-treatment continuum.

**Conclusions:**

The VELUT addresses methodological gaps in global mental health research by helping to position RCTs of MHPSS interventions along the IOM promotion-to-treatment continuum.

## Introduction

The Institute of Medicine (IOM) framework classifies mental health interventions into different categories, including promotion, prevention and treatment (Institute of Medicine, 2009). Numerous randomized controlled trials (RCTs) and systematic reviews have assessed the efficacy and effectiveness of promotion, prevention and treatment interventions for mental health across diverse populations (Barbui *et al.*, 2020; Hoare *et al.*, 2021; Papola *et al.*, 2020; Purgato *et al.*, 2023; van Zoonen *et al.*, 2014).

Promotion focuses on empowering individuals to enhance their well-being and resilience, and can be a distinct strategy or part of prevention and/or treatment efforts (Eaton, 2019). Prevention aims to avert, reduce or delay the onset of mental disorders through universal, selective or indicated approaches. Prevention may involve early detection and diagnosis, targeting risk and protective factors and reducing the impact of disease on functionality and quality of life (e.g., relapse prevention) (Acarturk *et al.*, 2022; Augustinavicius *et al.*, 2023; Buntrock *et al.*, 2017; Purgato *et al.*, 2021a, 2021b; Riello *et al.*, 2021; Purgato *et al.*, 2025a, 2025b). Treatment targets individuals with existing mental disorders, aiming to alleviate symptoms and improve functioning (Eaton, 2019; Tol *et al.*, 2015).

However, distinguishing between promotion, prevention and (early) treatment trials remains challenging for at least four reasons (Cuijpers, 2022). First, prevention studies require outcome measures that entail a clear ascertainment of the onset of a new disorder in adequately sized samples. However, this is lengthy, resource-intensive and often unfeasible, especially in low-resource settings. Many trials rely on proxy outcomes of incidence, such as symptom worsening, which can obscure the true effectiveness of prevention efforts and conceptually overlap with how treatments are commonly evaluated in RCTs. Second, binary classification of mental health is challenging and may oversimplify its complexity, as mental health exists more on a continuum rather than a strict healthy/ill dichotomy (Papola and Patel, 2025; Patel *et al.*, 2018). The chosen outcome measure may not necessarily be informative about whether the preventive potential of an intervention was investigated in the study or not. Third, studies may simultaneously focus on promotion, prevention and treatment. For several mental health and psychosocial support (MHPSS) interventions tested in RCTs, the boundaries between promotion, prevention and treatment are blurred, as all of them may be represented to different degrees. Moreover, the contents, components, levels, actors and possible mechanisms of action of MHPSS are only seldom explicitly described in many RCTs (Papola *et al.*, 2024; Purgato *et al.*, 2021a). This implies issues related to the proverbial black box, poor unpacking of the interventions’ components, and no explicit reference to an a priori theory of change (Miller *et al.*, 2021). Intervention manuals are often not reported or lack details on whether the intervention being tested was designed for promotion, prevention or treatment (Cuijpers *et al.*, 2024; Watts *et al.*, 2020). Fourth, having clarity on the aim of an intervention being evaluated is important for future use and implementation. If the distinction between promotion, prevention and treatment is diffuse, it might reflect uncertainty about the ultimate purpose of the intervention, and thereby its future use. Fifth, the reporting of many RCTs may be suboptimal, limiting appraisal of internal and external validity, and, therefore, inferential interpretation of findings. Sampling procedures are often poorly reported, and the inclusion and exclusion criteria of participants are often unclear (Miller *et al.*, 2023). This makes it difficult to draw coherent lines between populations at risk of and with mental disorders. For example, the same intervention given to the former population may be conceived as preventive, while for the latter as treatment.

Promotion, prevention and treatment RCTs should be clearly discerned to facilitate a better understanding of research findings in the field of public mental health. Appropriately appraising RCTs not only facilitates better overall evidence synthesis but also serves as a crucial tool for enhancing knowledge translation and policy uptake. A clear distinction can finally help researchers optimize the choice of outcomes, pinpoint research gaps, allocate efforts and resources where needed most, and avoid redundancies.

Against this background, we set out to develop a new measurement tool – the VErona-LUgano Tool (VELUT) – designed to position RCTs of MHPSS interventions along the promotion-to-treatment continuum through critical appraisal. This paper describes the methodological process, statistical analyses and results involved in the development and preliminary validation of this tool. It also provides the tool itself, along with instructions on how to use it in evidence synthesis.

## Methods

The protocol for this study was approved by the Ethics Committee of Università della Svizzera Italiana (CE_2024_04 of 25-01-2024), and has been published (Purgato *et al.*, 2023).

The methods, processes and procedures described here are adapted from the practical guide for the development of health measurement scales (David and Norman, 2015), which we complemented with elements of the Child Health and Nutrition Research Initiative research prioritization method (CHNRI) (Rudan, 2016; Rudan *et al.*, 2017; Shah *et al.*, 2016). The VELUT is an outcome-based tool composed of questions with appropriate answer options. The items were devised, drafted, selected and tested in multiple rounds.

**Figure 1.**
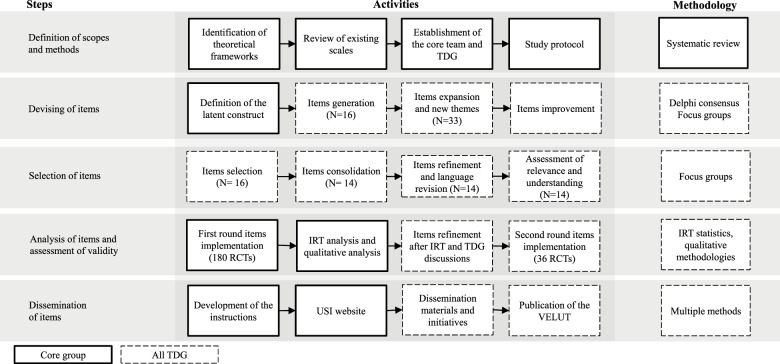
Study flowchart.

Legend: IRT: item response theory; N: number of items; RCT: randomized controlled trial; TDG: tool development group; USI: Università della Svizzera italiana; VELUT: Verona Lugano tool.

The development of VELUT began with a conceptualization step, in which we established a tool development group (TDG) composed of international experts (*n* = 14) in public mental health, global mental health and complementary disciplines, i.e., statistics and epidemiology. The TDG members are from 10 universities in 9 countries and are all co-authors of the present paper. The two coordinators of the TDG are the first co-authors of this paper (M.P. and E.A.) and worked in close collaboration with the last author, who originally conceived this endeavour (C.B.). Figure 1 presents the flowchart of the VELUT development methodological steps, described in detail below.

## Devising items

The TDG adopted an iterative process to define the construct(s) targeted for assessment and agreed to use both the Population, Intervention, Comparison and Outcome (PICO) framework and the IOM mental disorders preventive intervention research framework for devising, reviewing and selecting items (Institute of Medicine, 2009; Richardson *et al.*, 1995).

The TDG generated an initial pool of items related to, and collectively reflecting, the conceptualized constructs. We used two complementary approaches for item generation. First, we systematically searched for existing measures already developed to position RCTs along the promotion-to-treatment continuum; we found no studies reporting similar tools in any health field (Purgato *et al.*, 2023). Second, the TDG integrated multiple sources to develop the items, including theoretical frameworks, research expertise in the design and conduction of RCTs of MHPSS interventions, and collaboration with practitioners. Qualitative methods were employed with the TDG to elicit additional themes and insights regarding the usability and relevance of our tool. The qualitative methods included focus groups with TDG members, the tool’s intended end users, and three interview rounds focused on each individual item, its intended meaning, and exploration of its relevance and understanding. We also identified and discussed key methodological features of RCTs relevant to assessing the study’s promotion, prevention or treatment nature.

## Selection of items

First, TDG members categorized all devised items according to the PICO elements, carefully considering the potential of each item to inform the promotion, prevention or treatment nature of the RCTs domains according to the IOM framework (Institute of Medicine, 2009). Second, the two TDG coordinators (M.P., a clinical psychologist, and E.A., an epidemiologist) applied techniques adapted from the CHNRI research prioritization methodology (Rudan, 2016; Rudan *et al.*, 2017) to consolidate, combine and remove redundant items, maintaining an overall balance between the construct’s granularity and overall salient features. Third, M.P., E.A. and C.B. discussed and refined the wording and clarity of items. TDG members first participated in survey rounds to provide quantitative ratings on each item and their pertinence concerning the measured constructs (1 = not pertinent at all to 5 = extremely pertinent) and clarity (1 = extremely unclear to 5 = extremely clear). Fourth, TDG members participated in cognitive interviews to ensure consistency in the interpretation of the items (and response options) and to discuss face and content validity aspects. Finally, item wording and phrasing were improved through a consolidation step based on iterative discussion in a dedicated online session of TDG members, and independently revised by an external English mother-tongue for grammar and lexicon. The ensuing set of items formed the alpha version of the VELUT, which was used to assess a selection of 180 RCTs from recently published systematic reviews to support further item selection through IRT modelling (below).

## Statistical analysis

### Data collection

A team of seven evaluators (Appendix, page 8) used the tool to examine its application in a database of published RCTs on the effectiveness of MHPSS interventions (Papola *et al.*, 2020; Purgato *et al.*, 2023), which had been previously searched, selected and stored in a repository at the University of Verona.

Two assessors independently evaluated all 180 RCTs with the 16-item, alpha version of the VELUT, for the item response theory (IRT) modelling (described below) (MacCallum *et al.,* 2006; van der Linden, 2018; van der Linden and Hambleton, 1997). Next, we computed an overall score for each primary study based on the data obtained with the application of the VELUT, and then we prepared lists of deleted/combined/rephrased items – informed by both the empirical application of the VELUT and according to IRT results – for further discussion with the TDG.

Throughout the research process, the TDG engaged in methodological discussions online and during a 2-day in-person workshop in Lugano (Switzerland), including using an additional method to categorize RCTs.

### Statistical analyses

We performed a formal psychometric evaluation to assess the VELUT’s reliability and validity as described below in this paragraph. This included an internal consistency analysis and the factor structure of the VELUT using IRT. Using the standardized version of Cronbach’s alpha (Guttman’s lambda-6), we assessed the internal consistency of the scale, the average inter-item correlation and the signal-to-noise ratio (Albanese *et al.*, 2020), and interpreted IRT model results to identify redundant or overlapping items.

### Item response theory

We explored potentially uninformative items by measuring the response clustering (i.e., response or item non-variance ≥60%). We used confirmatory factor analysis (CFA), consistent with IRT, to verify whether the items fit the anticipated domain structure of the VELUT (i.e., PICO and IOM framework, above). Graded response (IRT) models for non-binary answer options were applied to relate the responses to our underlying construct of interest. This approach enabled us to explore characteristics of items, including their correlation with other items (e.g., discrimination) and relative location along the promotion-to-treatment continuum (e.g., item locations or thresholds). To summarize item loadings and thresholds, we generated Item Characteristic Curves (ICCs) to display the probability of endorsing a given response as a function of the latent trait level (i.e., the promotion-to-treatment continuum, on the *x*-axis), modelled with a cumulative logistic distribution. The ICCs visually summarize item location and discrimination parameters (Orlando *et al.*, 2000; van der Linden and Hambleton, 1997).

We used graded response logistic models (GRM) (Gross *et al.*, 2014), allowing both location and discrimination to vary across items, with the slope of the ICC depicting the ability to distinguish between neighbouring levels of the latent trait (David and Norman, 2015). We used the multidimensional IRT methods, specifically bifactor modelling (Reise, 2012) to capture possible departures from unidimensionality of the VELUT scale, and we explored goodness of fit of these models with Akaike information criteria, the Bayesian information criteria (Maydeu-Olivares and Joe, 2006), and the M2 statistics. The latter, which follows an approximate chi-square distribution, was purposely designed to work well with categorical item data (Maydeu-Olivares and Joe, 2005). M2 is less sensitive to sample size, it is computationally efficient, and it performs well even in the case of sparse data (i.e., a high number of response patterns). Our analysis of item fit was evaluated using normalized residuals (Bollen, 1989). Global model fit of IRT models was evaluated with the root mean square error of approximation (RMSEA), Standardized Root Mean Square Residual (SRMR) and Comparative Fit Index (CFI).

For all items, we calculated the item information function, which refers to the precision of the item in measuring the latent trait. Item information sums to a Test Information Function, which depicts the combined coverage and precision of the scale items relative to the promotion-to-treatment continuum (Lord, 1980). We statistically tested whether unidimensionality and local/conditional independence assumptions were met using parallel analysis with scree plots. Items that violated these assumptions were flagged for potential exclusion and discussed by the TDG. In addition, we evaluated the number of dimensions covered.

Finally, we used IRT for a preliminary validation study of the final version of the VELUT with 36 RCTs, reusing a subset of the 180 trials, that is, the 20% at each decile between the 10th and 90th percentiles of the original distribution of the latent trait. We applied the IRT GRM to evaluate the VELUT’s psychometric properties and generated ICCs, threshold distribution plots (TDPs) and test characteristic curves (TCCs). ICCs and TDPs provide a visualization of the location and discrimination parameters, the latent trait range covered by the items, and the response options thresholds. TCCs illustrate the precision of the VELUT in estimating the underlying latent trait (theta) at different points along the trait continuum, and how well the scale captures the latent trait (theta) across RCTs, with Marginal Reliability thresholds ≥0.90 interpreted as indicative of excellent reliability (Roth andTannenbaum, 2004). Analyses were carried out using Stata 15 (2017).

## Results

### Devising items

The TDG collaboratively generated an initial pool of 18 items and progressively added 15 more, integrating multiple sources reflecting the PICO and IOM frameworks. The comprehensiveness (i.e., content validity) of the provisional list of 33 items was confirmed by achieving thematic saturation for the key design and conduct features of RCTs that may provide information on the relevant aspects of the IOM construct.

### Selection of items and IRT results for the preliminary version of the VELUT

Of the 33 items developed, 6 were allocated to Population, 18 to Intervention, none to Comparison, five to Outcome in the PICO framework, and four to Study Setting (e.g., humanitarian, community). The items were evenly distributed across the IOM promotion, selective and indicated prevention and treatment continuum. The TDG members discussed the item list, eliminating redundant items (*n* = 3), rephrasing items (*n* = 17) and consolidating similar ones to maintain a balance between granularity and construct representation. We retained 16 items and improved their wording and clarity for the next steps.

The average ratings for pertinence and clarity of the 16 items, assessed by members of the TDG using a 1–5 Likert scale, were 3.76 (0.46, range: 2.85–4.37) and 3.75 (0.41, range: 3.16–4.67), respectively. Cognitive interviews confirmed that all TDG members understood all items and response options as intended and could interpret them correctly. Minor wording adaptations were made, such as the use of the term ‘screening’ instead of ‘selection’ for item 1, the use of the term ‘primarily’ instead of ‘initially’, the elimination of redundant words (i.e., ‘for example’), and the choice and clarification of abbreviations. Distributions of response options for the rated 180 RCTs based on the preliminary version of the VELUT are reported in the appendix (Appendix Table e1). Items u1 and u2; u2 and u3; u13, u14 and u16 were discussed with the TDG as their implementation was challenging according to the reviewers during the RCT assessment. The correlation matrix confirmed a strong correlation between items u1 and u2; u2 and u3; and between u13, u14 and u16. As a result, and after the conceptual in-person TDG discussion, some items were rephrased, one item (‘Were subgroup analyses done based on risk profiles and/or symptoms?’) was moved to the beginning of the tool to provide contextual information about the RCT being rated instead of providing information along the promotion-to-treatment continuum, and Item u16 (‘In this specific RCT, was the trial setting humanitarian?’) was deleted. Some other items were combined. Principal component analysis and CFAs, respectively, indicated one strong factor explained 57% of the total variance and 84% of the shared inter-item variance, while parallel analysis with a scree plot suggested strong statistical evidence for unidimensionality. Cronbach’s α of 0.94 suggested strong internal consistency reliability. The very poor fit of a Rasch model (RMSEA = 0.329; CFI = 0.92; SRMR = 0.313) requires better model specification. A 2-p graded-response IRT had better fit to data (RMSEA = 0.158; CFI = 0.98; SRMR = 0.076), and was much improved with a bifactor solution (RMSEA = 0.100; CFI = 0.99; SRMR = 0.059) that allowed specific factors to explain intercorrelations between p1, p2, p3 and o1, o2 in addition to common covariation explained by the common factor. The ICCs of the 16 items showed high heterogeneity of both item difficulty and slope (i.e., discrimination parameter) (Appendix Figure e4), with partially overlapping thresholds (u13 and u14) likely due to low or no endorsement of given response categories (Appendix Figure e5). The ICCs highlighted potentially redundant items (u6 and u11, which were highly correlated, *r* = 0.96) (Appendix Figure e6).

Further item selection led to minimal depreciation of the test information plot (Appendix Figure e7), and through intensive, iterative discussion during the in-person workshop, the TDG agreed that eight items were either duplicates, not informative or not applicable. Six were deleted, 2 combined and 1 was moved to the beginning of the VELUT and transformed into a classification question, not contributing to the overall score. An agreement was reached about the remaining eight items, their phrasing and respective response options, and a recognition to allow missing data on items in the form of missing/unclear information from the published RCT.

### Validation study and final version of the VELUT

Table 1 reports the frequencies of responses to the eight items of the final version of the VELUT applied to 36 RCTs independently selected by the TDG psychiatric epidemiologist (AG). To prioritize representation of trials along the full promotion-to-treatment continuum in this restricted set of 36 RCTs, we selected 20% of trials at each decile between the 10th and 90th percentiles of the original distribution of 180 trials. This selection of trials ensured a decent spread of responses across all items, with the expected exception of the two outcome-related items (O1 and O2) for which answer options of indicated prevention prevailed. There were 12 ‘unclear’ responses out of 36 to item P3 regarding participants’ symptomatology at baseline, which was treated as missing data in graded-response IRT models and handled assuming missingness was random conditional on other variables in the model (e.g., MAR). All item discriminations (estimated via weighted least squares means and variance adjusted estimation) were high (>0.58) to very high (>0.93), indicating a high correlation between the VELUT items and the underlying latent promotion-to-treatment continuum of interest.

**Table 1.** Frequencies of responses from the application of the VELUT to 36 selected RCTs, and item parameters

	Responses (*N*)					Item discrimination (loadings)	Item difficulty (thresholds)^a^		
	Promotion/Univ. prevention (−3)	Sel. prevention (−1)	Ind. prevention (+1)	Treatment (+3)	Unclear or insufficient information^a^	WLSMV estimator, delta parameterization	−3, −1	−1, 1	1, 3
**Population**									
P1. Did study participants go through a screening process to be enrolled in the study?	5	17	5	9	0	0, 90	−2, 49	0, 65	1, 55
P2. How was the screening conducted?	6	14	7	7	2	0, 89	−2, 03	0, 49	1, 79
P3. Were the study participants symptomatic at baseline based on an optimized cut-off?	4	7	6	7	12	0, 87	−1, 93	−0, 21	1, 10
**Intervention**									
I1. How was the intervention originally conceived and described, as outlined or implied in a manual, if available?	11	7	11	7	0	0, 92	−1, 28	0, 00	2, 16
I2. What was the timing of the intervention with respect to the exposure (s)?	3	14	10	4	5	0, 89	−2, 86	0, 27	2, 48
I3. In this specific RCT, what was the primary aim of the intervention?	7	6	16	7	0	0, 93	−2, 37	−0, 98	2, 37
**Outcome**									
O1. Which was the considered outcome?	4	2	21	9	0	0, 58	−1, 50	−1, 19	0, 83
O2. How was the considered outcome measured?	4	2	22	8	0	0, 65	−1, 61	−1, 27	1, 01

Loadings: The strength of the relationship between an item and the latent trait. Threshold: Points on the latent trait (θ) where the probability of selecting a specific response category or higher is 50%. WLSMV: weighted least squares means and variance adjusted for categorical data, such as Likert-scale responses.

a Thresholds are on the latent trait scale, not on the observed scale.

**Figure 2.**
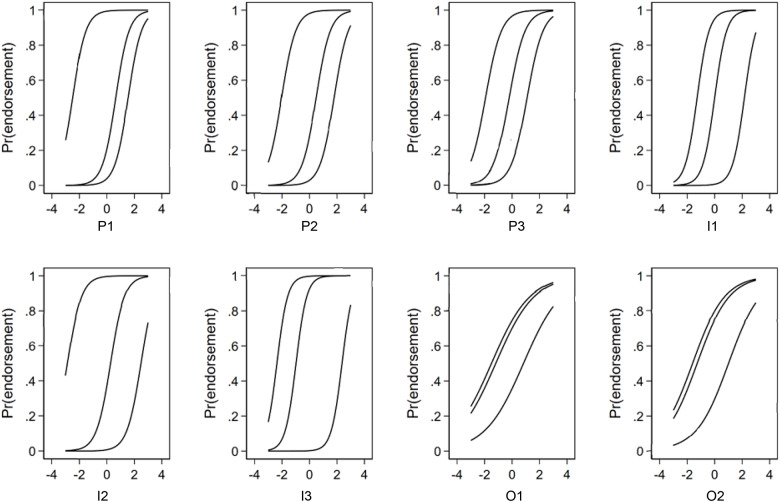
Item characteristic curves.

The thresholds between response options (i.e., category boundaries) across all items ranged between corresponding theta values of −2.86 to 2.48, with most intermediate thresholds falling close to 0 on the latent trait continuum (assumed to be standard normally distributed [N{0,1}]) (Table 1). Figure 2 presents the ICC of the final version of the VELUT. The steepness of slopes (i.e., discrimination) of the ICC shown in the figure supports the ability of each item to distinguish between neighbouring levels of the underlying latent trait, the promotion-to-treatment continuum. ICCs show the broad range of location parameters across items.

**Figure 3.**
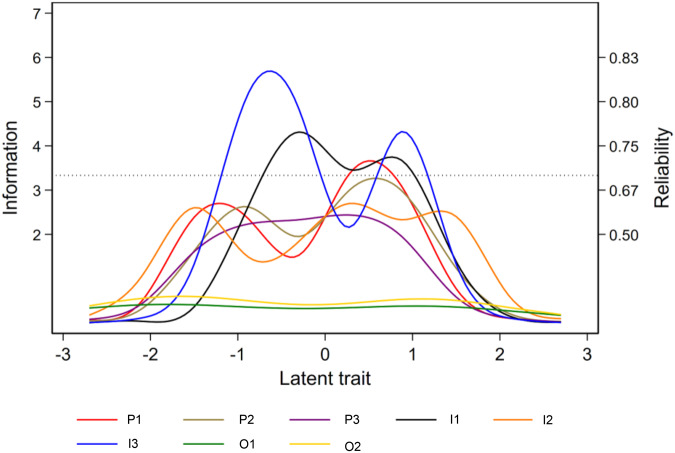
Item information function (Mplus).

**Figure 4.**
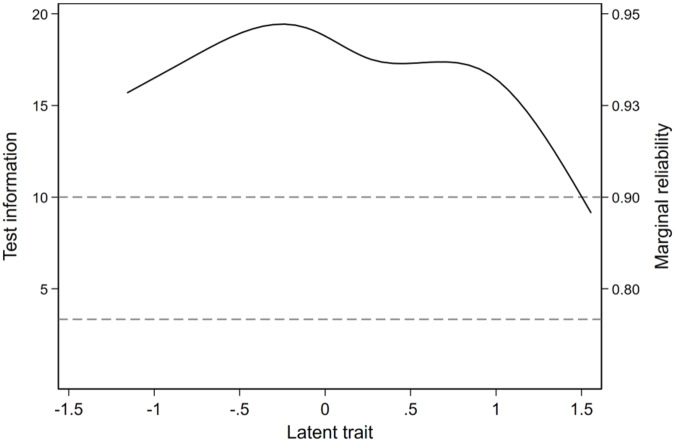
Test information function (Mplus).

The information and reliability of each item and the overall VELUT scale are illustrated in Figures 3 and 4, respectively.

**Figure 5.**
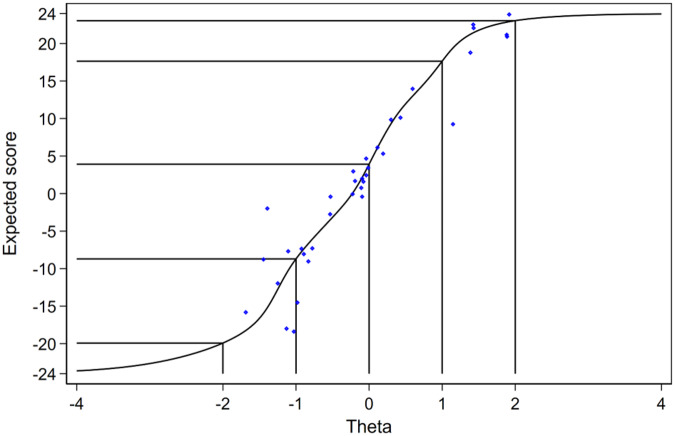
Test characteristic curve (TCC).

The item information functions (Fig. 3) show high information for all items across a broad range of theta, except for O1 and O2. The test information function suggests that the VELUT is a strong measure of the latent trait across all the RCTs appraised (Fig. 4). Test information corresponding to a marginal reliability >0.90 was present across the entire observed range of the promotion-to-treatment latent trait, indicating excellent reliability of the scale, that is, a highly precise measurement of theta. The slope of the TCC (Fig. 5) is steep, indicating that the VELUT should differentiate well between RCTs with varying levels of promotion-to-treatment trait, and the linear relationship with the computed score suggests that the VELUT scores predictably and consistently increase as the latent trait does. Flat areas of the TCC were confined to extreme score values (Fig. 5). This result supports the good interpretability of the VELUT overall scores and their correspondence with the promotion-to-treatment continuum.

After extensive in-person discussion in the workshop, the TDG agreed that the overall VELUT score may be complemented with the computation of the frequencies of response options that correspond to the IOM domains of promotion, selective prevention, indicated prevention and treatment to recognize that one RCT can provide evidence for each domain, irrespective of its positioning across the promotion-to-treatment continuum. Both computational combinations of the responses can be used to inform the classification of RCTs in evidence synthesis (see https://www.iph.usi.ch/it/strumenti/velut). The VELUT tool is presented in Table 2.

**Table 2.** The Verona-Lugano Tool – VELUT

Preliminary questions	Response options
1. Indicate the outcome considered of this specific appraisal	Open question
2. Indicate the analytical sample of this specific appraisal	1. Whole analytical sample
	2. Subsample
2b. Please describe the subgroup	Open question
**Item**	**Label (score)**
**P1. Did study participants go through a screening process to be enrolled in the study?^a^**	
All target individuals in the source populations are eligible and included (no selection/screening made)	Promotion/Univ. prevention (−3)
Inclusion criteria were based on exposure to risk factors	Sel. prevention (−1)
According to inclusion/ exclusion criteria, the planned study sample comprises participants with symptoms, but below a diagnostic threshold/clinical cut-off	Ind. prevention (+1)
According to inclusion/ exclusion criteria, the planned study sample comprises participants with a formal diagnosis/above diagnostic threshold/clinical cut-off	Treatment (+3)
Unclear or insufficient information	NA
**P2. How was the screening conducted?^b^**	
No screening, or participants enrolled irrespective of screening/selection results	Promotion/Univ. prevention (−3)
With a risk factors assessment measure(s) (e.g., having a migration background, currently unemployed, currently pregnant)	Sel. prevention (−1)
With a scale(s) for symptoms or functional impairment (with optimized cut-off; e.g., scoring above a threshold for psychological distress, or being distressed but scoring below a threshold indicating a clinical diagnosis)	Ind. prevention (+1)
With a diagnostic tool(s) (e.g., positivity on structured clinical interview: SCID, MINI, Hamilton, etc.), or scoring above a symptom scale threshold indicating a clinical diagnosis	Treatment (+3)
Unclear or insufficient information	NA
**P3. Were the study participants symptomatic at baseline based on an optimized cut-off?^c^**	
All participants below a diagnostic threshold	Promotion/Univ. prevention (−3)
A minority of participants above a diagnostic threshold/ clinical cut-off	Sel. prevention (−1)
Most participants above a diagnostic threshold/ clinical cut-off	Ind. Prevention (+1)
All participants above a diagnostic threshold/ clinical cut-off	Treatment (+3)
Unclear or insufficient information	NA
**I1. How was the intervention originally conceived and described, as outlined or implied in a manual, if available?**	
Intervention primarily conceived for promotion/universal prevention (according to theory of change) (for example, empowerment and/or information, education, skill development and behavioural changes)	Promotion/Univ. prevention (−3)
Intervention primarily conceived for selective prevention (for example, mainly about addressing or mitigating risk factors)	Sel. prevention (−1)
Intervention primarily conceived for indicated prevention (for example, mainly about Stress-management; problem solving)	Ind. prevention (+1)
Intervention primarily conceived for treatment (for example, structured psychotherapy with a manual, or treatment)	Treatment (+3)
Unclear or insufficient information	NA
**I2. What was the timing of the intervention with respect to the exposure(s)?**	
Intervention delivered to all independently from any exposures/events and/or contents of the intervention appropriate for the general population, regardless of if and when people were exposed to any event	Promotion/Univ. prevention (−3)
Intervention delivered before/during the exposure/event and/or contents focused on coping mechanisms (or support, for example, housing or job hunting) for normal life stressors, and/or in preparation for potential future consequences of the event	Sel. prevention (−1)
Intervention delivered after the exposure/event but before the onset of the mental health condition	Ind. prevention (+1)
Intervention delivered after the exposure/event and after the onset of the mental health condition (assessed as either part of the study or independently)	Treatment (+3)
Unclear or insufficient information	NA
**I3. In this specific RCT, what was the primary aim of the intervention?**	
To improve overall wellbeing (physical, psychological, social)	Promotion/Univ. prevention (−3)
To target modifiable risk factors, social determinants or exposures	Sel. prevention (−1)
To reduce subclinical symptoms and/or distress/to prevent the development of a condition	Ind. prevention (+1)
To treat a specific condition	Treatment (+3)
Unclear or insufficient information	NA
**O1. Which was the considered outcome?**	
Well-being/knowledge and understanding/‘change in attitudes’ or similar outcome	Promotion/Univ. prevention (−3)
Risk factors or protective factors	Sel. prevention (−1)
Subclinical symptoms level or mental health condition onset	Ind. prevention (+1)
Clinical symptoms level or mental health condition progression	Treatment (+3)
Unclear or insufficient information	NA
**O2. How was the considered outcome measured?**	
Scale for wellbeing/knowledge and understanding/‘change in attitudes’ or similar outcome	Promotion/Univ. prevention (−3)
Measure of risk profile/level	Sel. prevention (−1)
Checklist (with or without cut-off) or diagnostic tool to measure subclinical symptoms and/or to determine caseness	Ind. prevention (+1)
Clinical scale for reduction of clinical symptoms/severity (cut-off)	Treatment (+3)
Unclear or insufficient information	NA

NA, unclear or insufficient information.

a The study population screened for the presence of a mental conditions or indicators and proxies of psychological distress or other mental health condition using a mental health instrument/symptom checklist.

b Consider the operationalization of the inclusion and exclusion criteria (if any).

c See study sample characteristics, typically reported in Table 1.

## Discussion

This paper presents the development and preliminary psychometric validation of the VELUT, a novel measurement tool designed to position RCTs along the IOM continuum, ranging from mental health promotion to treatment. Tool development followed rigorous, iterative qualitative and quantitative psychometric procedures and involved international experts from complementary disciplines, who participated as members of an interdisciplinary TDG.

Through multiple rounds of item generation, refinement meetings, qualitative feedback, cognitive interviews and statistical analyses, we identified a final set of eight items demonstrating strong psychometric properties. Our findings indicate that the VELUT exhibits excellent internal consistency, high item discrimination and robust unidimensionality, supporting its construct validity in assessing the promotion-, prevention- or treatment focus of MHPSS interventions in global and in public mental health.

Results from IRT analyses revealed that the category thresholds for all eight items were widely spaced, indicating good item distribution and strong discrimination across varying levels of the latent trait representing the spectrum from promotion to treatment. All 8 items were found to be non-redundant and informative, ensuring comprehensive coverage of the underlying construct. The development of the VELUT addresses a critical gap in global mental health research by providing a structured, evidence-based approach to characterize intervention studies along a continuum that is frequently conceptually and methodologically ambiguous.

Many of our study RCTs were classified as indicated prevention, a finding that aligns with prevailing trends in the scientific literature, which focus on population groups already presenting symptoms of mental disorders (Cuijpers, 2003, 2022; Thom *et al.*, 2024).

In general, conducting prevention research in mental health is not easy. It requires measuring the incidence of mental disorders to determine whether an intervention reduces the onset of disorders. This presents three distinct challenges. First, the accurate measurement of incidence in prevention trials requires delivering a formal diagnostic interview, a time-consuming process which necessitates the involvement of trained professionals compared to using self-assessed symptom scales (Dattani, 2023; Jensen-Doss and Hawley, 2010). Prevention trials require substantial financial resources and access to trained professionals capable of administering diagnostic assessments. These resources can be difficult to mobilize, particularly in low- and middle-income settings where mental health services are limited, and mental health professionals are scarce. Second, mental health prevention research often requires a broad and diverse participant base and long-term follow-up periods (Legemaat *et al.*, 2023). Mental health disorders often emerge gradually and can be influenced by several factors, including the structural and social determinants of mental health (Lund *et al.*, 2018; Machado *et al.*, 2024; Rose-Clarke *et al.*, 2020). Also, prevention research often targets at-risk populations, complicating recruitment efforts and introducing potential ethical considerations and constraints (World Health Organization, 2022). Third, boundaries between the different levels of prevention are often blurred in mental health research, may be subjectively disputed and interpreted. This is part of the conceptual challenge that guided the development of the VELUT.

The VELUT is a new tool in mental health. There are several critical appraisal tools of primary studies in the scientific literature (Medicine CEBM, 2023) conceptually different from the VELUT. For example, it shares several premises with the Cochrane Risk of Bias tool (RoB) (Higgins *et al.,* 2023). Like the RoB, the VELUT is outcome-specific and transparent. However, while the Cochrane RoB is explicitly designed to evaluate the RoB and the internal validity of primary studies, focusing primarily on methodology, the VELUT provides a complementary classification, that is a structured approach for positioning RCTs along the IOM continuum from promotion to treatment, as well as for quantifying the extent to which each RCT emphasizes promotion/universal prevention, selective prevention, indicated prevention or treatment. This quantification, which no existing tools currently offer, is important, as 2 RCTs may have the same overall VELUT score yet exhibit markedly different profiles across each IOM domain. This mitigates potential misclassification bias and underscores the conceptual limitation of relying on aggregate scores alone. Consequently, no fixed threshold was imposed for categorizing RCTs based on total scores, and we recommend against the use of cutoffs that would be inherently arbitrary and may introduce bias.

Our study has limitations. First, our methodology is novel. However, to our knowledge, there are no established methodologies to develop a scale in evidence synthesis. Our methodology is grounded on existing CHNRI methodology, and considers two robust conceptual frameworks, namely PICO and IOM. Second, different from the WHO guidelines development groups, though the TDG was gender balanced, low- and middle-income countries were less represented than high-income countries. However, several members of the TDG have a long experience in humanitarian, resource-poor settings, which may have helped to account for some attention to socio-cultural factors in all the steps of the VELUT development process. We followed a balanced, multidisciplinary, highly participatory, democratic and replicable approach. Third, although we did confirm that the unidimensionality assumption of the IRT model was met, we cannot exclude deviations from local independence of items, although this seems unlikely. A strength of this study is that we used IRT models to inform item selection and to embed a preliminary validation conducted on a large set of relevant RCTs assessed by a trained assessment team. A further strength worth noting is that we also confirmed the known groups’ validity of the VELUT cherry-picking a few RCTs at each extreme of the spectrum, where VELUT scores were lowest and highest, respectively. This provides further empirical support for the construct validity of this new tool.

The TDG has developed a new pragmatic, free and accessible tool to address key challenges in evidence synthesis. Moreover, the VELUT aims to also promote better RCT design by introducing a public mental health perspective to existing methodological tools and resources. The VELUT helps to systematically – and in a pragmatic, user-friendly way – clarify which methodological characteristics align a primary study with a focus on promotion, prevention or treatment. This classification can be done with an overall VELUT score complemented by the computation of the frequencies of response options that correspond to the IOM domains of promotion, selective prevention, indicated prevention and treatment. This classification may guide key methodological decisions such as inclusion and exclusion criteria, outcome measures and intervention selection. The VELUT may also allow the classification of RCTs and, accordingly, guide subgroup and sensitivity analyses in systematic reviews and meta-analyses. Additionally, it will orient policymakers and funders when considering the criteria for RCTs to be funded or implemented in specific global and public health initiatives.

The VELUT has been specifically developed to address gaps in global mental health research and evidence synthesis. But its conceptual framework and structure lend to broader applicability across other areas of health research that may also share the challenge of misclassification of studies along the promotion to treatment continuum. For example, the VELUT can be applied to trials involving children and adolescents, as well as populations with severe mental conditions or comorbidities, since the items are designed to capture general trial features independent of age group or diagnostic complexity. This new tool has potential implications for the design of new clinical trials that could be more precisely focused on studying promotion, prevention or treatment. For example, inclusion and exclusion criteria, tools for outcome assessment and analyses and implementation settings could be defined using the VELUT. Implications also regard the clinical practice and the use of the evidence in the intervention choice; priority setting and strategic planning activities, and, last but not least, the development of calls for application and evaluation research applications for funding agencies and policy makers.

## Supporting information

10.1017/S2045796025100280.sm001 Purgato et al. supplementary material Purgato et al. supplementary material

## Data Availability

Data will be made available upon motivated request to the contact authors, and after discussion with the TDG.

## Supplementary material

The supplementary material for this article can be found at https://doi.org/10.1017/S2045796025100280.
