# Supplementary material for: How to distinguish promotion, prevention, and treatment trials in public mental health: development and validation of the VErona-LUgano Tool (VELUT)
Source: Epidemiol Psychiatr Sci. 2025 Nov 12;34:e54. doi: 10.1017/S2045796025100280 (PMC12646188; doi:10.1017/S2045796025100280)

**Index**

[Instructions for applying the VELUT 2](#_Toc195256755)

[Table e1. Distribution of response options for 180 RCTs 2](#_Toc195256756)

[Table e2. Correlation matrix for the 16-item version of the VELUT 3](#_Toc195256757)

[Figure e2. Scree plot with parallel analysis from PCA *(principal component analysis)* 4](#_Toc195256758)

[Figure e3. Generic IRT model 4](#_Toc195256759)

[Figure e4. Item characteristics curves of the preliminary version of the VELUT 5](#_Toc195256760)

[Figure e5. Threshold distribution plot for the preliminary version of the VELUT 5](#_Toc195256761)

[Figure e6. Item Information Curves of the preliminary version of the VELUT 6](#_Toc195256762)

[Figure e7. Item Information Curves of the preliminary version of the VELUT 7](#_Toc195256763)

[Description of the training for the assessment team 7](#_Toc195256764)

Instructions for applying the VELUT

| **Preliminary questions** | **Response options** |
| --- | --- |
| **Insert study ID as follows: First author + year (of publication)** | Open question |
| **1. Indicate the outcome considered of this specific appraisal** | Open question |
| **2. Indicate the analytical sample of this specific appraisal** | 1. Whole analytical sample  2. Subsample |
| **2b. Please describe the subgroup** | Open question |

Table e1. Distribution of response options for 180 RCTs

 

Table e2. Correlation matrix for the 16-item version of the VELUT: Results based on 180 RCTs

Figure e2. Scree plot with parallel analysis from PCA: Results based on 16 items, N=180 RCTs

**PCA:** *principal component analysis*

Figure e3. Generic IRT model of 16 items

**
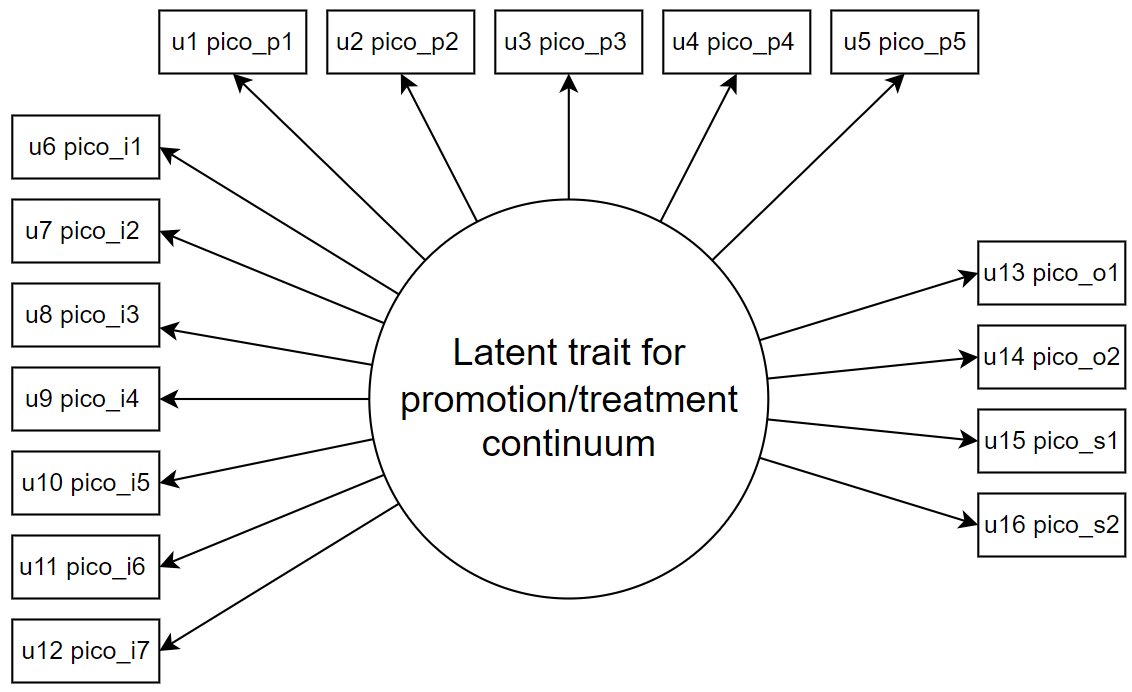
**

Figure e4. Item characteristics curves of the preliminary version of the VELUT: Results based on 16 items, N=180 RCTs

Figure e5. Threshold distribution plot for the preliminary version of the VELUT: Results based on 16 items, N=180 RCTs


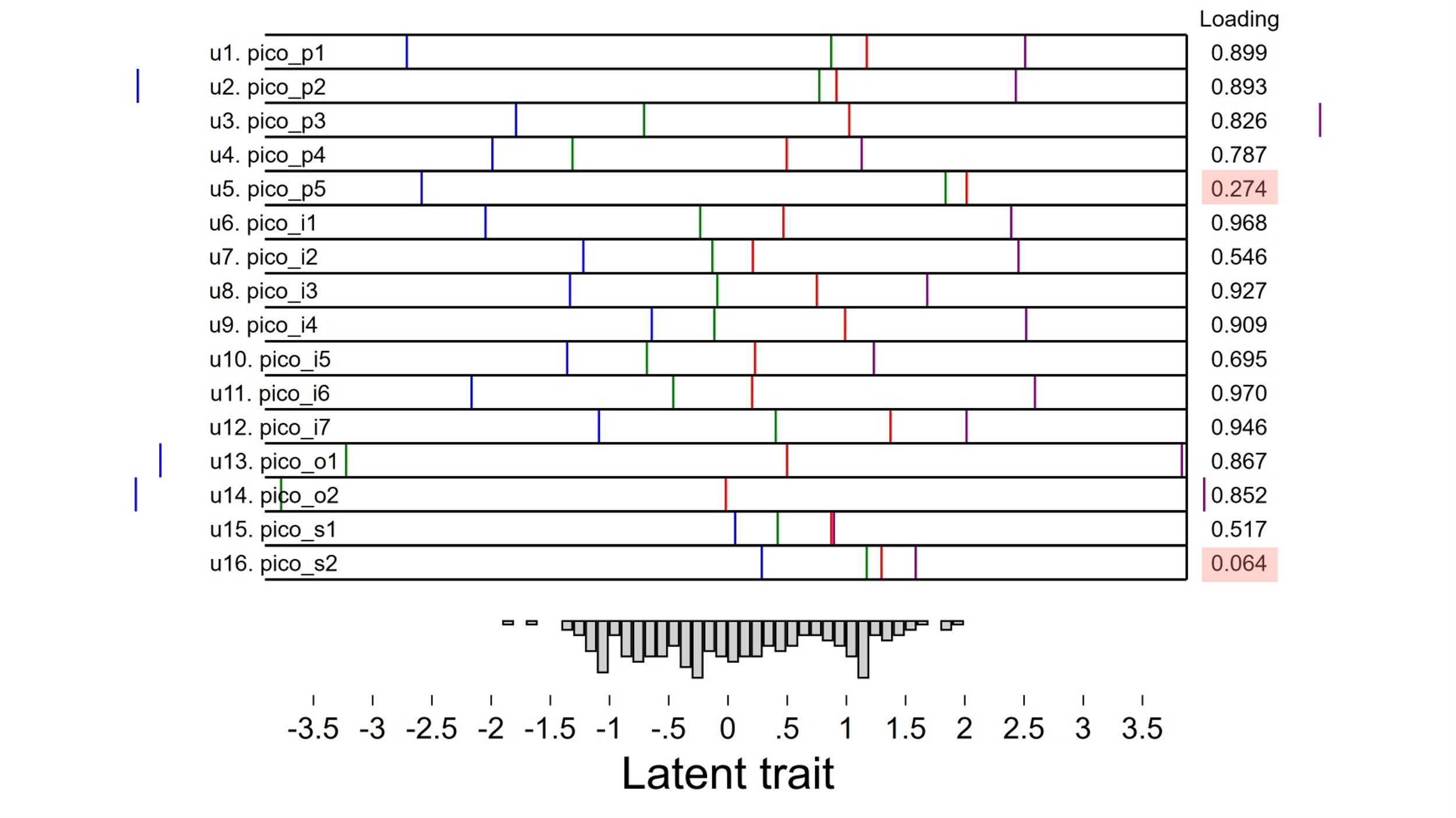


Figure e6. Item Information Curves of the preliminary version of the VELUT: Results based on 16 items, N=180 RCTs


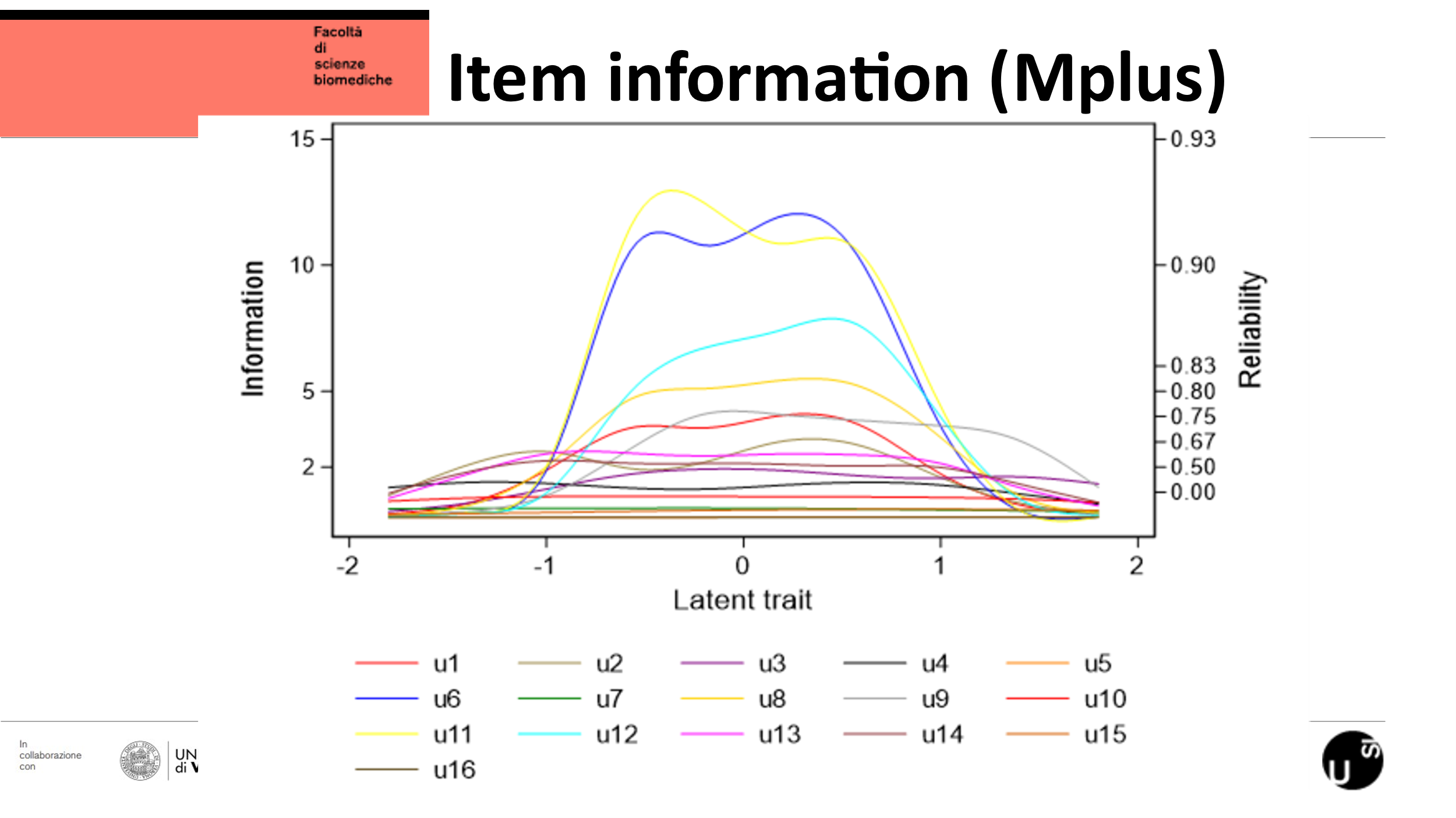


Figure e7. Test Information Curve of the preliminary version of the VELUT: Results based on16 items, N=180 RCTs


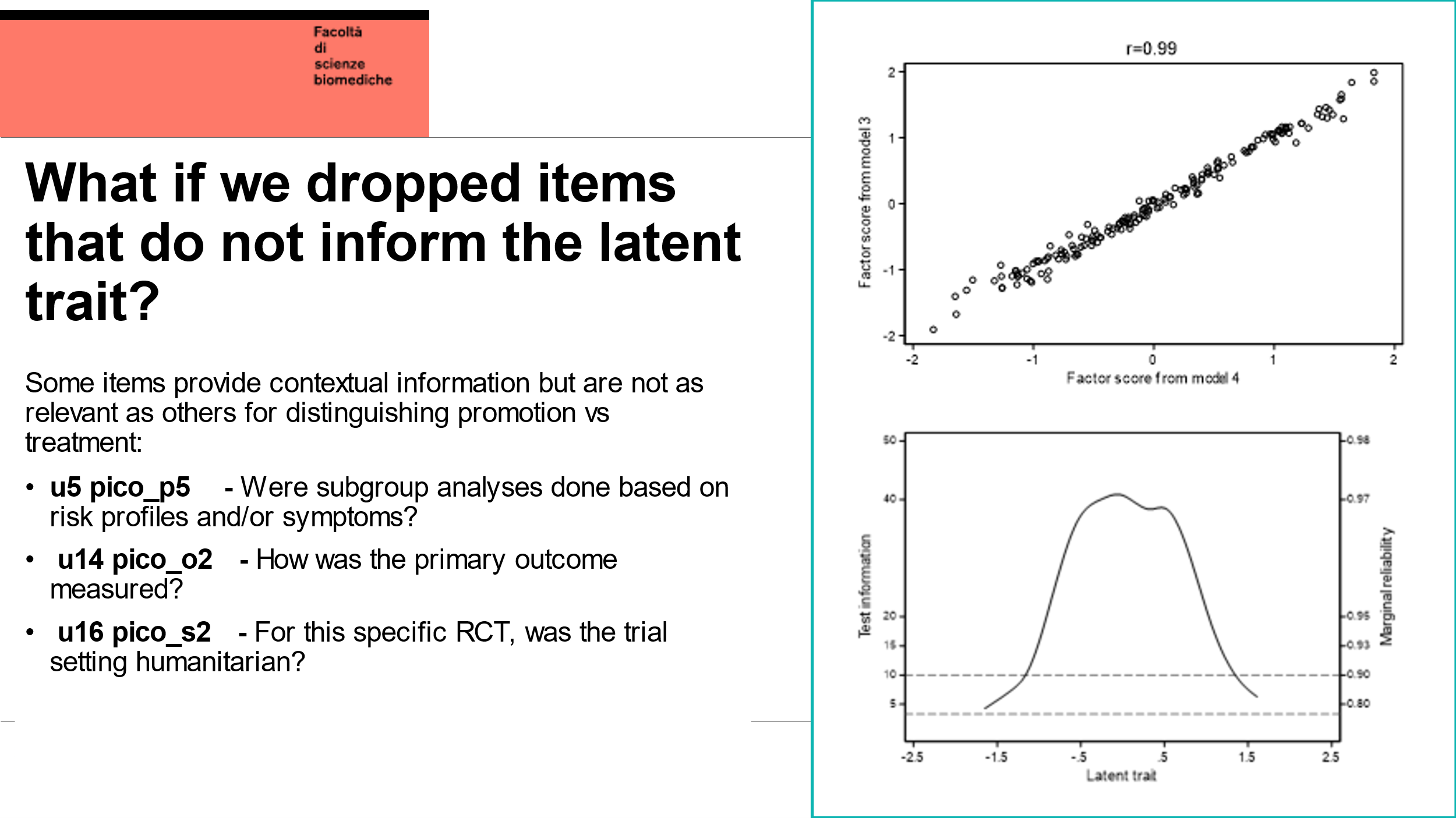


Description of the training for the assessment team

A training activity was conducted by MP and EA in hybrid format, to equip junior researchers with the skills needed to apply the VELUT for categorizing primary studies within the promotion-to-treatment continuum. The training was composed of two sessions with additional on-demand supervisions (approximately one supervision per week for three months). Junior researchers were introduced to the tool’s framework, including its criteria for classification, methodological considerations, and practical examples.

The training included a hands-on component where participants worked with a set of diverse primary studies. In pairs, they systematically applied the tool, discussing classification decisions and addressing challenges in categorization. MP and EA guided them through discrepancies, emphasizing reliability and consistency in applying the criteria.

To reinforce learning, participants engaged in a calibration exercise, independently categorizing a set of studies before comparing results in a plenary discussion. Differences were analyzed to refine their understanding and ensure alignment in applying the tool.

The training concluded with reflections on the challenges encountered with specific RCTs and/or items. By the end of the training, junior researchers demonstrated confidence in using the tool and a clear understanding of its role in mapping primary studies. The senior staff of our team did not receive a specific training, but detailed instructions on the application of the tool.

Below is a graphical representation of the training activities for the application of the VELUT.


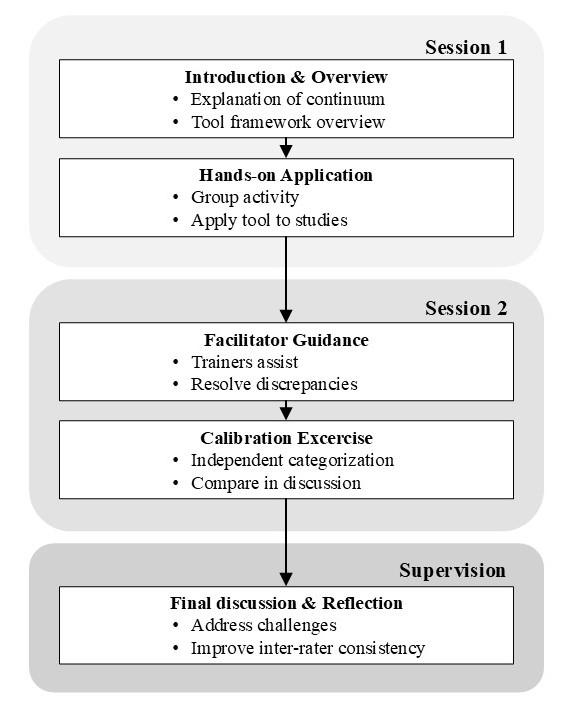

Supplement: Purgato et al. supplementary material [file S2045796025100280sup001.docx]
